# Natural diversity of potato (*Solanum tuberosum*) invertases

**DOI:** 10.1186/1471-2229-10-271

**Published:** 2010-12-09

**Authors:** Astrid M Draffehn, Sebastian Meller, Li Li, Christiane Gebhardt

**Affiliations:** 1 Max-Planck Institute for Plant Breeding Research, Carl von Linné Weg 10, 50829 Köln, Germany

## Abstract

**Background:**

Invertases are ubiquitous enzymes that irreversibly cleave sucrose into fructose and glucose. Plant invertases play important roles in carbohydrate metabolism, plant development, and biotic and abiotic stress responses. In potato (*Solanum tuberosum*), invertases are involved in 'cold-induced sweetening' of tubers, an adaptive response to cold stress, which negatively affects the quality of potato chips and French fries. Linkage and association studies have identified quantitative trait loci (QTL) for tuber sugar content and chip quality that colocalize with three independent potato invertase loci, which together encode five invertase genes. The role of natural allelic variation of these genes in controlling the variation of tuber sugar content in different genotypes is unknown.

**Results:**

For functional studies on natural variants of five potato invertase genes we cloned and sequenced 193 full-length cDNAs from six heterozygous individuals (three tetraploid and three diploid). Eleven, thirteen, ten, twelve and nine different cDNA alleles were obtained for the genes *Pain-1*, *InvGE*, *InvGF*, *InvCD141 *and *InvCD111*, respectively. Allelic cDNA sequences differed from each other by 4 to 9%, and most were genotype specific. Additional variation was identified by single nucleotide polymorphism (SNP) analysis in an association-mapping population of 219 tetraploid individuals. Haplotype modeling revealed two to three major haplotypes besides a larger number of minor frequency haplotypes. cDNA alleles associated with chip quality, tuber starch content and starch yield were identified.

**Conclusions:**

Very high natural allelic variation was uncovered in a set of five potato invertase genes. This variability is a consequence of the cultivated potato's reproductive biology. Some of the structural variation found might underlie functional variation that influences important agronomic traits such as tuber sugar content. The associations found between specific invertase alleles and chip quality, tuber starch content and starch yield will facilitate the selection of superior potato genotypes in breeding programs.

## Background

Invertases are ubiquitous enzymes that irreversibly cleave sucrose into the reducing sugars fructose and glucose. Plant invertases not only play an important role in the partitioning of carbon between source tissue (photosynthetic leaves) and heterotrophic sink tissues such as seeds, tubers and fruits, they also function in plant development and in responses to biotic and abiotic stress. Three types of invertase isoenzymes, which are encoded by small gene families, are regularly found in plants. Cell wall-bound acidic invertases cleave sucrose in the apoplastic space (apoplastic invertases). Soluble acid invertases are located in the vacuole (vacuolar invertases), whereas soluble neutral invertases are located in the cytoplasm [1,2].

In the potato (*Solanum tuberosum*), carbon is stored as starch polymers in tubers. Besides starch, tubers also contain small amounts of sucrose, glucose and fructose. The amounts of starch and sugars present in tubers depend on the genotype and on environmental factors. Storage at low temperature (e.g. 4°C) for several weeks leads to conversion of a small fraction of starch into sugars in tubers, with consequent accumulation of glucose and fructose, in particular [3,4]. This phenomenon of 'cold-induced sweetening' is an adaptive response to cold stress, as sugars have long been known to have an osmoprotective function in plants [5]. Invertases, together with other proteins, play a role in determining the tuber sugar content before and during cold storage. Invertase activity is present in tubers and increases during cold storage [6-8]. Transcripts of vacuolar invertase accumulate in the tubers upon cold storage [9-11] and invertase antisense inhibition changes the hexose to sucrose ratio in the tubers [10]. The content of the reducing sugars glucose and fructose in tubers is an important criterion of quality for the potato processing industry. During deep frying at high temperatures, reducing sugars and amino acids undergo a non-enzymatic Maillard reaction, which results in a dark brown color and inferior taste of potato chips or French fries due to polyphenol formation [12,13]. With increasing tuber sugar content, the chip color changes from light yellow to brown or even black. Although the enzymatic and biochemical steps in the interconversion between starch and sugars are well known in plants in general and potato in particular, the triggering and the regulation of cold-induced sweetening in potato is not fully understood [3,4,14]. In addition, the impact of natural variation in potato genes involved in carbohydrate metabolism on the quantitative variation of tuber starch and sugar content among different genotypes is completely unknown.

Genetic mapping of quantitative trait loci (QTL) for tuber starch and sugar content on the one hand [15,16] and localization of genes that function in carbohydrate metabolism and transport on the other [17] have pointed to a number of candidate genes, which roughly colocalize with QTL for tuber starch and sugar content [18]. Among these are three independent loci encoding invertase genes. Potato cDNAs encoding apoplastic and vacuolar invertases have been cloned and characterized previously [10,11,19,20]. Using invertase cDNA sequences as molecular markers, these three potato invertase loci have been mapped [17]. The *Pain-1 *locus on chromosome III encodes a vacuolar invertase, whereas the loci *Inv*_*ap*_*-a *and *Inv*_*ap*_*-b *on chromosomes X and IX, respectively, encode apoplastic invertases [17]. Two tandemly duplicated genes, *InvGE *and *InvGF*, encoding apoplastic invertases have been identified in one genomic fragment of 9 kb [21]. *InvGE *and *InvGF *are orthologous to the closely related tomato invertase genes *LIN5 *and *LIN7*, respectively, which are also tandemly duplicated and located on tomato chromosome 9 [22]. The *Inv*_*ap*_*-b *locus maps to the orthologous position on potato chromosome IX. In view of the colinearity of the genomes of potato and tomato [23], *InvGE/InvGF *can both be assigned to the *Inv*_*ap*_*-b *locus. The locus *Inv*_*ap*_*-a *on chromosome X was mapped with the same cDNA probe 'pCD141' [20] as *Inv*_*ap*_*-b*, and is orthologous to a tomato locus on chromosome 10 encoding the tandemly duplicated invertase genes *LIN6 *and *LIN8 *[22]. Genomic sequences of the potato *Inv*_*ap*_*-a *and *Pain-1 *loci have not been reported.

Association mapping in populations of tetraploid potato varieties and breeding clones has revealed 'single-strand conformation polymorphisms' (SSCPs) [24] in invertase genes at all three loci, which were associated with tuber starch content, and/or chip color [25,26]. Most significant were associations with SSCP markers derived from the *Pain-1 *gene on chromosome III [25]. These marker-trait associations are either direct (i.e. allelic variants of the invertase gene itself are causal for the phenotypic variation) or indirect (genes that are physically linked but unrelated to the invertase gene are responsible for the QTL) in effect. In the latter case, the association observed at an invertase locus is the result of linkage disequilibrium between the invertase gene and other, unknown genes in the same haplotype block [27]. Unfortunately, neither QTL linkage mapping down to single-gene resolution [28] nor high-resolution association mapping using thousands of individuals for complex traits such as tuber starch content and chip color is practicable in the cultivated potato. An alternative approach is the direct functional analysis of invertase allelic variants to elucidate their roles in determining variation in tuber starch and sugar content. This requires the cloning and characterization of full-length invertase cDNA alleles from representative potato genotypes, and the identification of cDNA alleles that correspond to the associated SSCP markers. Here we report the results of such a study.

## Methods

### Plant material

Invertase alleles were cloned from the tetraploid cultivars Satina, Diana and Theresa, and from the diploid *S. tuberosum *lines H82.337/49 (P18), H80.696/4 (P40) and H81.839/1 (P54) [29]. The tetraploid genotypes were selected from 34 varieties included as standards in the association mapping population 'ALL' described in [25], because they possess invertase markers that are associated with tuber starch content (TSC), starch yield (TSY), and chip quality in autumn after harvest (CQA) and after cold storage (CQS) (Table 1). The diploid genotypes were the parents of the mapping populations used to map cold-sweetening QTL [16]. Plants were grown in pots in the greenhouse (day temperature 20-24°C; night temperature 18°C; additional light from 6 am to 9 pm) or in a Saran-house under natural light and temperature conditions from May to September. Leaves and flowers were harvested throughout the growing season. Tubers were harvested from mature plants and stored at 4°C in the dark. Genomic DNAs from 219 members of the association mapping population ALL were used for SNP genotyping. This population consists of 34 standard varieties and 209 breeding clones from three potato breeding companies. The ALL population has been phenotyped for tuber yield (TY, [dt/ha]), starch content (TSC, [%]), starch yield (TSY, [dt/ha]), and chip quality after harvest in autumn (CQA, score from 1 to 9) and after cold storage at 4°C (CQS, score from 1 to 9) [25].

**Table 1:** Presence/absence in cvs. Satina, Diana and Theresa of invertase markers associated with tuber traits.

Locus	Chromosome	Marker fragment	Association with	Polarity of effect	Satina	Diana	Theresa
*Pain-1*	III	*Pain1-9a *^1, 3^	TSC, TSY, CQA, CQS	↑	yes	yes	no

		*Pain1-8c *^1, 3^	TSC, TSY, CQA, CQS	↑	yes	yes	no

		*Pain1-5c *^1, 3^	TSC, TSY, CQA, CQS	↑	no	yes	no

		*Pain1-5d *^3^	TSC	↑	yes	no	no

		*Pain1-5b *^3^	TSC, CQS	↓	no	no	yes

*Inv*_*ap*_*-b*	IX	*InvGE-6f *^2, 4^	CQA, CQS	↑	yes	yes	yes

		*InvGF-4d *^2, 5^	CQA, CQS	↑	yes	yes	yes

*Inv*_*ap*_*-a*	X	*pCD141-3c *^3^	TSC, CQA, CQS	↓	yes	no	no

^1 ^SSCP markers *Pain1-9a*, *Pain1-8c *and *Pain1-5c *are in strong linkage disequilibrium with each other [25]

^2 ^Markers *InvGE-6f *and *InvGF-4d *are in nearly complete linkage disequilibrium with each other [26].

^3 ^SSCP (single strand conformation polymorphism) marker [25].

^4 ^SCAR (sequence characterized amplified region) marker [26].

^5 ^ASA (allele specific amplification) marker [26].

### RNA extraction and cDNA synthesis

Total RNA was extracted from leaves and flowers using the ToTally RNA Isolation Kit (Ambion, Cambridgeshire, UK) following the supplier's protocol. Total RNA was extracted from tuber tissue powdered in liquid nitrogen, using the Plant RNA Isolation Kit from Invitrogen (Karlsruhe, Germany) following the supplier's protocol. Tuber RNA was further purified by high-salt precipitation to remove polysaccharides and by lithium chloride precipitation to remove low-molecular-weight RNA. The RNA solution was adjusted to 1 mL by adding RNase-free water, mixed with 250 μl isopropanol and 250 μl high salt solution (1.2 M sodium citrate, 0.8 M NaCl) and incubated on ice for 2 h. RNA was recovered by centrifugation at 13,000 rpm for 30 min at 4°C. The pellet was rinsed with 70% ethanol, and centrifuged at 13000 rpm for 5 min at 4°C. After removing the ethanol, the pellet was air-dried and dissolved in RNase-free water at a minimum concentration of 200 ng total RNA per μl. High-molecular-weight RNA was precipitated by mixing with 0.5 volumes of 5 M LiCl and incubating on ice overnight at 4°C. RNA was collected by centrifugation as above, rinsed with 70% ethanol, dried and dissolved in 20-50 μl RNase-free water depending on pellet size. All RNA samples were further purified using the DNA-*free*™ Kit (Ambion). RNA concentration and quality were analyzed by measuring the A_260 nm_/A_280 nm _(1.8 - 2.0) and A_260 nm_/A_230 nm _(2 - 3) ratios using a Nanodrop ND-1000 spectrophotometer (Peclab, Erlangen, Germany). RNA integrity was tested on 1% agarose gels loaded with 300-500 ng of total RNA. Total RNA was stored at -80°C. First-strand cDNA was synthesized according to the supplier's protocol from 2.0 μg of total RNA, using 200 U of Superscript™ II Reverse Transcriptase (Invitrogen) per reaction and 500 ng of oligo(dT)_16-18 _(Roche, Mannheim, Germany) as primers. First-strand cDNA was treated with RNase H (Roche, Mannheim, Germany) for 20 min at 37°C. First-strand cDNA (1 μl per reaction) was then used for allele amplification and cloning.

### Invertase cDNA allele amplification, cloning and sequencing

Primers spanning the start and stop codons of the invertase genes (Table 2) were designed based on the sequences of GenBank accession numbers L29099, X70368 (*Pain-1*), AJ133765 (*InvGE *and *InvGF*), Z21486 (*InvCD111*) and Z22645 (*InvCD141*). *Pain-1 *alleles were amplified using as template first-strand cDNA from tubers stored for 25 days at 4°C. *InvGE *and *InvGF *alleles were amplified from first-strand cDNA templates obtained from leaves and flowers. *InvCD111 *and *InvCD141 *alleles were amplified from leaf cDNA templates. Oligonucleotides were purchased from Invitrogen (Karlsruhe, Germany), Sigma-Aldrich Chemie (Taufkirchen, Germany) and Operon Biotechnologies (Köln, Germany). Polymerase chain reactions (PCR) (annealing temperatures 55-65°C, 30-50 cycles) were performed using the Fast Start High Fidelity PCR System (Roche, Mannheim, Germany) or KOD Hot Start DNA Polymerase (Novagen, Darmstadt, Germany) according to the supplier's protocols. PCR products were purified with the High Pure PCR Purification Kit (Roche, Mannheim, Germany) and ligated into the pGEM^®^-T/T Easy vector (Promega, Mannheim, Germany) following the supplier's protocols. Competent cells of *E. coli *strains DH5α and DH10B (MAX Efficiency^® ^DH5α™ and ElectroMAX™ DH10B™ competent cells from Invitrogen, Karlsruhe, Germany) were transformed with recombinant plasmids [30]. Transformed strains were cultured according to standard methods [31]. Plasmid DNA was isolated with Plasmid Isolation Mini or Midi Kits (Qiagen, Hilden, Germany) and sequenced by the DNA Core Facility at the Max-Planck Institute for Plant Breeding Research on Applied Biosystems (Weiterstadt, Germany) ABI PRISM 377, 3100 and 3730 sequencers, using BigDye terminator (v3.1) chemistry. Premixed reagents were from Applied Biosystems. SNPs were identified in multiple sequence alignments (http://multalin.toulouse.inra.fr/multalin/multalin.html). Due to the large number of cDNAs sequenced, most variants were represented at least two times in independent PCRs primed with first-strand cDNA from the same genotype. cDNA alleles were then defined based on the consensus sequences of all clones obtained from an individual genotype. In some cases, the number of full-length cDNA sequences per genotype was low (Table 3). Eleven alleles (*InvGE-Db, InvGE-Sb, InvGF-Te, InvGF-Sb, InvCD141-Sa, InvCD141-Dd2, InvCD141-Td2, InvCD111-Sb, InvCD111-Sc, InvCD111-Ta, InvCD111-P54d*; see Tables S3, S4, S5 and S6 in additional files 1, 2, 3 and 4) were therefore defined based on a single cDNA sequence.

**Table 2:** PCR primers used for cDNA allele cloning and amplicon sequencing, product sizes, annealing temperatures.

Gene	Ampli-con	Forward (F) and reverse (R) primers 5' to 3'	Length [bp]	**T**_**a **_**[C°]**
*Pain-1*	cDNA	F: ATGGCCACGCAGTACC R: GATGAATTACAAGTCTTGCAAGGG	1920	55

	Exon 1	F: ATGGCCACGCAGTACC R: GTTGAAAATGGTAAGCAGTTC	360	52

	Exon 3	F: CACAAGGGATGGTATCATC R: CCCATCCCTTCTGCAG	861	51

	Exon 7	F: CACTCAATTGTGGAGAGCTTTG R. CAAGTCTTGCAAGGGGAAGG	201	59

*InvGE*	cDNA	F: ATGGAATTATTTATGAAAAGCTCTTCTCTTTGGGGGT R: TTAGTGCATCTTAGGTACATCCATGCTCCAAGC	1761	55

	Exon 1	F: GCTCTTCTCTTTGGGGTTTAG R: TTAGGAGGTTGAAAATGAAAAC	199	56

	Exon 6	F: GATAACTCAGTAGTGGAGAGTTTTG R: GTGCATCTTAGGTACATCCATG		56

*InvGF*	cDNA	F: ATGGATTATTCATCTAATTCTCGTTGGGCTTTGCCAG R: TCAATATTGTATCTTAGCTTTGCCCATACTCCATGC	1743	55

*InvCD141*	cDNA	F: ATGGAGATTTTAAGAAGATCTTCTTCTCTTTGGGTT R: CTAGTGCAACTTTGCATTAGCCATGCTCCAAGC	1746	55

	Exon 3	F: GGTCCAATGTATTACAATGGAG R: GCAACTGTGATTCCTTTGATTTC	1023	56

	Exon 4	F: GAAGTGATTTTCTCATTCACAAG R: CTTGAGGCATCAGAACACATAAG	246	56

*InvCD111*	cDNA	F: ATGGATTGTTTAAAAAAGTCTTCTC R: TCAATAAGAAGAGTGACCAAATGACCAATTCA	1767	55

**Table 3:** Summary of invertase cDNA allele cloning and SNP identification.

	No. of cDNA alleles identified per genotype (No. of full-length clones sequenced)						Total number	No of different alleles (nucleic acid sequence)	No of different amino acid sequences	No of SNP's identified	No of amino acid changes
Gene	Satina	Diana	Theresa	P40	P54	P18					

*Pain-1*	2 (9)	3 (16)	2 (8)	2 (8)	1 (6)	2 (7)	12 (54)	11	6	78	35

*InvGE*	4 (10)	3 (8)	4 (19)	2 (8)	2 (5)	2 (9)	17 (59)	13	12	137	53

*InvGF*	4 (14)	2 (4)	2 (4)	2 (4)	2 (10)	1 (2)	13 (38)	10	9	97	26

*InvCD141*	3 (6)	2 (5)	3 (6)	1 (2)	2 (4)	2 (5)	13 (28)	12	11	102	32

*InvCD111*	3 (5)	1 (1)	2 (4)	1 (1)	2 (3)	0 (0)	9 (14)	9	8	65	36

Total number	16 (44)	11 (34)	13 (41)	8 (23)	9 (28)	7 (23)	64 (193)	55	46	479	182

### Invertase genomic sequences

The BAC (bacterial artificial chromosome) libraries BA and BC, both constructed from high-molecular-weight genomic DNA of the diploid, heterozygous genotype P6/210 and arrayed on high density filters, were screened by filter hybridization with labeled probes for cDNAs *Pain-1 *[10] and *pCD141 *[20] as described [32,33]. Positive BACs were confirmed by gene-specific PCR using primers as described above and Southern gel-blot hybridization. Complete BACs were custom sequenced by Eurofins MWG Operon (Ebersberg, Germany) using a 454 platform [34]. In addition, the genes *Pain-1 *and *InvCD141 *were custom sequenced (GATC Biotech, Konstanz, Germany) by primer walking on the BACs using the dideoxy chain-termination method [35]. Sequencing of the *Pain-1 *gene by primer walking was performed on the BAC selected for complete sequencing, whereas the gene *InvCD141 *was sequenced using BAC BC37c23. BAC sequences were annotated using the Apollo Genome Annotation and Curation Tool, version 1.9.8 [36].

### Phylogenetic tree construction

Phylogenetic trees were generated using the maximum parsimony method based on a Clustal W amino acid alignment [37] of all invertase sequences integrated in the MEGA 4 software [38]. In all, 1000 bootstrapping runs were performed to obtain an estimate of the reliability of each branchpoint.

### SNP genotyping

Amplicons were generated from genomic DNAs of the heterozygous individuals of the ALL population with locus-specific primers (Table 2). The amplicons were purified with ExoSAP-IT^® ^(USB, Cleveland, USA) and custom sequenced at the Core Facility for DNA Analysis of the Max-Planck Institute for Plant Breeding Research. The dideoxy chain-termination sequencing method was employed using an ABI PRISM Dye Terminator Cycle Sequencing Ready Reaction Kit and an ABI PRISM 3730 automated DNA Sequencer (Applied Biosystems, Weiterstadt, Germany). SNPs were identified by sequence alignment and visual examination of the sequence trace files for overlapping base-calling peaks. In each tetraploid individual bi-allelic SNPs were assigned to one of five allelic states (two homozygous and three heterozygous). The SNP allele dosage in heterozygous individuals (1:3, 2:2 or 3:1) was estimated from the relative heights of the overlapping base-calling peaks, both manually and with the Data Acquisition and Analysis Software DAx (Van Mierlo Software Consultancy, Eindhoven, The Netherlands). Pyrosequencing [39] was carried out on a PSQ 96 system (Biotage AB, Uppsala, Sweden) using the PSQ 96 SNP Reagent Kit according to the manufacturer's protocol. For pyrosequencing of the *Pain1_SNP1544 *alleles, the following primers were used to generate an amplicon of 252 bp: Forward : 5'-GGACCATTTGGTGTCGTTGT-3', reverse: 5'-(biotin)TCTTCCTCCTTGAGCAAAGC-3'. The sequencing primer was 5'-CGTTGTAATTGCTGATCA-3'.

### Haplotyping

Within the SATlotyper (v.1.0.5) software [40] the SAT solver MiniSat_v1.14_cygwin was used to model haplotypes from unphased SNP data scored in the ALL population. Individuals with missing data for one or more SNPs in the set chosen for haplotyping and individuals with suboptimal quality of the amplicon sequence were excluded from haplotype analysis.

### Association test

SNPs were tested for association with the phenotypic values using the general linear model (GLM) procedure in SPSS 15.0 (SPSS GmbH Software, Munich, Germany). The model used was

y*=origin+marker+error

where y* stands for the adjusted phenotypic means [25]. *Origin *is a factor (fixed) with four classes to identify the origin of each genotype in the ALL population from one of three breeding companies or from the standard varieties [25]. *Marker *is a factor (fixed) with five levels, corresponding to the five SNP allele dosages: 0 for allele absent, 1, 2, 3 and 4 for allele present in simplex, duplex, triplex or quadruplex dosage. Population structure has been evaluated and described in [25].

## Results

### Genomic structure of the invertase loci *Pain-1 *and *Inv*_*ap*_*-a*

**Figure 1:**
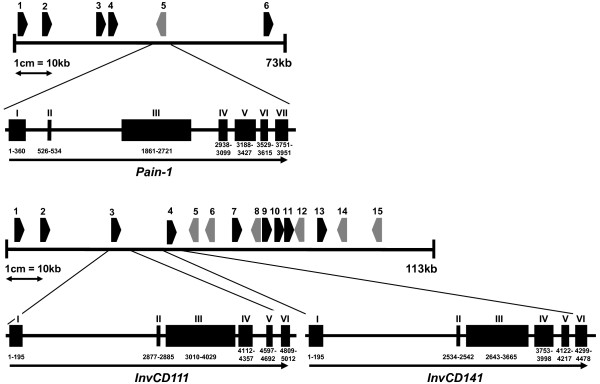
**Structure of the *Pain-1 *locus on potato chromosome III (**A**) and the *Inv-ap-a *locus on chromosome X (**B**)**. Annotated open reading frames (ORFs) are numbered as in Table S1 in additional file 5. Transcriptional orientation is indicated by arrowheads. Left to right transcripts are shown in black, right to left transcripts in grey. The intron/exon structures of *Pain-1 *(ORF 6 on BAC BC149o15), *InvCD111 *and *InvCD141 *(ORFs 3 and 4 on BAC BC163l15) are shown as blow-ups.

Whereas the genomic organization of the tandemly duplicated genes *InvGE *and *InvGF *at the *Inv*_*ap*_*-b *locus on chromosome IX is already known [21], no genomic sequences of the loci *Pain-1 *and *Inv*_*ap*_*-a *have been reported. We therefore isolated, sequenced and annotated the BAC clones BC149o15 (HQ197978) and BC163l15 (HQ197979), which were selected from BAC libraries based on cross-hybridization with *Pain-1 *and *InvCD141 *probes. In addition to 454 sequencing of whole BACs, the genes *Pain-1 *and *InvCD141 *were also sequenced by the dideoxy chain-termination method and primer walking. BC149o15 contained one full-length copy of the *Pain-1 *gene. The *Pain-1 *sequences obtained from the same BAC by two different sequencing techniques (454 and Sanger sequencing) differed by a six-nucleotide insertion in intron 2. The *Pain-1 *gene consists of seven exons and six introns and is around 4 kbp long (Figure 1). The BAC clone BC163l15 contained two tandemly duplicated invertase genes, *InvCD111 *and *InvCD141*, which corresponded to the cDNA clones pCD111 and pCD141 [20]. The two genes, 5 and 4.5 kbp in size, are separated by 7.3 kbp and each consists of six exons and five introns (Figure 1). Sanger sequencing of *InvCD141 *in a third BAC (BC37c23) revealed a gap of around 1 kbp in the assembly of the 454 sequences in intron 2. Besides that, the two sequences differed by five nucleotides. The full annotation of BACs BC149o15 and BC163l15 is shown in Table S1 in additional file 5. The individual genomic sequences of *Pain-1*, *InvCD141 *and *InvCD111 *are available as GenBank accessions HQ110080, HQ110081 and HQ197977, respectively.

### Natural diversity of Pain-1 cDNA alleles

Fifty-four full-length cDNA clones were sequenced from tubers of the tetraploid varieties Satina, Diana and Theresa, and the diploid genotypes P18, P40 and P54 that had been stored in the cold. Sequence comparisons identified eleven different cDNA alleles that translated into six amino acid sequences (Table 3). Fifty-eight single-nucleotide polymorphisms (SNPs) were detected when the eleven cDNA alleles were aligned. The inclusion of three soluble acid invertase sequences recovered from the NCBI database (accessions AAA50305 = *Stpain1_a *from cv Russet Burbank [11], ACC93585 = *Stpain1_c *from cv Kufri Chipsona and AAQ17074 = *Stpain1_b *from an unknown genotype) in the alignment uncovered sixteen additional SNPs. Sequencing of exons 1, 3 and 7 of *Pain-1 *in the 34 standard varieties included in the association mapping population ALL identified four further SNPs. The total of 78 SNPs included one tri-allelic SNP and resulted in amino acid changes at 35 positions, corresponding to 5.5% of the deduced *Pain-1 *protein sequence (Table 3, Table S2 in additional file 6, Figure S1 in additional file 7). Phylogenetic analysis of the nucleic acid sequences (not shown) separated the cDNA alleles into four similarity groups - *a*, *b*, *c *and *d*. The group *d *alleles from the diploid genotype P40 were most divergent from the others (see Table S2 in additional file 6). In order to identify cDNA alleles corresponding to the SSCP markers associated with the tuber traits (Table 1), and to detect any novel SNP-trait associations, we genotyped the ALL population for 15 SNPs in exon 3 of *Pain-1 *by amplicon sequencing, and for *SNP1544 *in exon 5 by pyrosequencing. These sixteen SNPs included diagnostic SNP alleles for groups *a*, *b*, *c *and *d *and for some individual alleles, i.e. one of the two alternative nucleotides was specific for an allele group or an individual allele (Table S2 in additional file 6). The SNP alleles C_552_, A_718_, A_1544 _and T_741 _were diagnostic for allele group *a*, A_528 _for group *b*, C_777 _and G_1068 _for group *c*, and T_591 _and G_637 _for group *d*. Five SNPs present in exon 3 of the cDNA alleles were not detected in the corresponding amplicon sequences (SNPs 534, 723, 834, 852, 927). Conversely, four additional SNPs absent in the cDNA alleles were detected and scored in the amplicon sequences of the ALL population (SNPs 639, 825, 888, 943). The best correspondence between presence/absence of SNP alleles and the associated SSCP markers in the ALL population was found for the SNP alleles in group *a *(Table 4). The SNP alleles C_552 _and A_718 _corresponded most closely to the SSCP marker *Pain1*-*8c*, A_1544 _to *Pain1-9a*, and the alleles T_741 _and C_1143 _were correlated with *Pain1-5d*. A_1544 _was also weakly correlated with *Pain1-5c. *None of the SNPs corresponded to SSCP marker *Pain1-5b*. The 16 SNPs were also tested for association with the tuber traits TSC, TY, TSY, CQA and CQS. SNP alleles C_552_, A_718 _and A_1544 _were positively associated with chip quality, tuber starch content and starch yield (lighter chip color, higher tuber starch content and starch yield, Table 5), as were the corresponding SSCP markers *Pain1*-*8c *and *Pain1-9a *[25]. The weak association of SSCP marker *Pain1-5d *with tuber starch content was confirmed by the corresponding SNP allele T_741 _(Table 5). The six genotypes used for cDNA cloning represent only a fraction of the genetic diversity of invertases in *S. tuberosum*. To obtain more comprehensive information on the number and frequency of *Pain-1 *haplotypes distributed in populations of tetraploid, heterozygous cultivars used in breeding programs, we selected eleven SNPs, which were diagnostic for allele groups *a *(SNPs 552, 718 and 1544), *b *(SNP528), *c *(SNPs 612 and 1068) and *d *(SNPs 612 and 637), a novel allele *x *not found among the cDNA clones (SNP 825), and the individual alleles *Sa *(SNP741), *P18b *(SNP1050) and *Stpain1-a *(SNP639 from cv Russet Burbank). Haplotypes were modeled using SATlotyper [40], a software that infers haplotypes from unphased SNP data in heterozygous polyploids. Fifteen haplotype models with frequencies higher than 1% were obtained based on eleven SNPs scored in 189 individuals of the ALL population (Table 6). The haplotypes *A*, *B *and *C *with frequencies higher than 10% accounted for 60% of all chromosomes in the population (4 × 189 = 756), whereas 35% were accounted for by 12 haplotypes with frequencies between 1% and 10%. Among the latter were five haplotypes that included the associated SNP alleles C_552_, A_718_, A_1544 _and T_741_. Five haplotype models were verified by corresponding cDNA clones, whereas the remaining ten haplotypes were novel (Table 6).

**Table 4:** Similarity of distribution in the ALL population between associated Pain-1 SSCP markers and Pain-1 SNP alleles.

	SNP alleles in group *a*					Control allele in group *c*
SSCP marker	C_552_	A_718_	A_1544_	T_741_	C_1143_	G_1068_

*Pain1-9a*	0.63 ^1^	0.59	**0.79**	0.29	0.30	0.20

*Pain1-8c*	**0.79**	**0.73**	0.54	0.32	0.33	0.16

*Pain1-5c*	0.36	0.32	0.50	0.07	0.06	0.17

*Pain1-5b*	0.01	0.01	0.00	0.02	0.01	0.34

*Pain1-5d*	0.47	0.51	0.44	**0.62**	**0.65**	0.07

^1 ^Jaccard similarity measure

**Table 5:** Associations of invertase SNP alleles with chip quality (CQA, CQS), tuber starch content (TSC) and/or starch yield (TSY).

Invertase SNP allele	Invertase allele or allele group	SNP allele frequency	**CQA *F ***^**1**^	CQS *F*	TSC *F*	TSY *F*
*Pain1- A*_*718 *_*(C*_*552*_*) *^*2*^	*a*	0.04	3.421* ↑	8.161*** ↑	8.344*** ↑	6.053** ↑

*Pain1- A*_*1544*_	*a*	0.06	ns	3.947* ↑	10.683*** ↑	5.656** ↑

*Pain1-T*_*741*_	*a*	0.03	ns	ns	2.649* ↑	2.923* ↑

*InvGE-A*_*85 *_*(A*_*86*_*)*	*a, d*	0.30	ns	ns	5.006** ↑	4.044** ↑

*InvGE-G*_*95 *_*(G*_*106*_*)*	*a*	0.06	ns	4.032* ↑	ns	ns

*InvCD141_T*_*543 *_*(A*_*280*_*, T*_*288*_*, T*_*339*_*, A*_*630*_*, C*_*1030*_*, G*_*1031*_*, T*_*1096*_*)*	*Sa*	0.14	5.615** ↓	3.850* ↓	6.125** ↓	ns

*InvCD141-G*_*765*_	*e*	0.27	ns	4.596** ↑	3.949** ↑	2.706* ↑

^1^*F *value; the *p *value is indicated as * (p < 0.05), ** (p < 0.01) or *** (p < 0.001); the arrow indicates the direction of the effect, upwards for a positive (better chip quality, higher starch content, higher starch yield), downwards for a negative effect of the SNP allele, respectively.

^2^SNP alleles shown in parentheses are in strong linkage disequilibrium with the allele for which the association has been shown, and therefore display similar associations.

**Table 6:** Pain-1 haplotype models obtained with Satlotyper.

Haplotype	**cDNA allele or group **^**1**^	Haplotype frequency	**SNP 528 (*b*) **^**2**^	SNP 552 (*a*)	SNP 612 (*c,d*)	SNP 637 (*d*)	SNP 639 (*Stpain1-a *)	SNP 718 (*a*)	SNP 741 (*Sa*)	SNP 825 (*x*)	SNP 1050 (*P18b*)	SNP 1068 (*c*)	SNP 1544 (*a*)
*A*	*P18b*	0.295	A	T	A	A	C	G	C	T	T	C	C

*B*	*b*	0.173	A	T	A	A	C	G	C	T	C	C	C

*C*	*c*	0.139	T	T	G	A	C	G	C	T	C	G	C

*D*		0.049	T	T	G	A	C	G	C	T	C	C	C

*E*		0.046	A	T	G	G	C	G	C	C	C	C	C

*F*		0.041	T	T	A	A	C	G	C	T	C	C	C

*G*		0.038	T	T	A	A	C	G	C	T	T	C	C

*H*	*d*	0.036	T	T	G	G	C	G	C	T	C	C	C

*I*		0.026	A	T	G	A	C	G	C	T	T	C	C

*K*		0.025	T	T	A	A	C	G	C	T	C	C	A*

*L*		0.024	T	T	G	A	C	G	C	T	C	G	C

*M*		0.018	A	C*	A	A	C	A*	C	T	T	C	C

*N*	*Sa*	0.017	T	C*	A	A	C	A*	T*	T	C	C	A*

*O*		0.014	T	C*	A	A	C	A*	C	T	C	C	C

*P*		0.013	A	T	A	A	C	G	T*	T	C	C	C

^1 ^cDNA allele or allele group that corresponds to the haplotype.

^2 ^cDNA allele or allele group, for which the SNP is diagnostic, see Table S2 in additional file 6.

Associated SNP alleles are highlighted by *.

### Natural diversity of InvGE and InvGF cDNA alleles at the *Inv*_*ap*_*-b *locus

Fifty-nine *InvGE *and thirty-eight *InvGF *full-length cDNAs were cloned from leaf and flower tissue of the three tetraploid and the three diploid genotypes (Table 3), and subsequently sequenced. In contrast to the reported flower-specific expression of *InvGF *[21], we found that *InvGF *was expressed also in leaves. The expression level in leaves was genotype dependent (data not shown).

Comparative sequence analysis of the *InvGE *cDNAs identified 13 different cDNA alleles encoding 12 amino acid sequences (Table 3, Tables S3 and S4 in additional files 1 and 2). Alignment of the *InvGE *cDNAs and *InvGE *from accession AJ133765 (cv Saturna, *StinvGE-c*) [21] identified 133 SNPs (two of them tri-allelic) and two insertions/deletions (indels) of one codon each. Sequencing of the amplicons of exons 1 and 6 in the 34 standard varieties uncovered two additional SNPs. The 135 SNPs plus the two indels resulted in 53 amino acid changes, corresponding to 9.1% of the deduced *InvGE *protein sequence (Figure S2 in additional file 8). Grouping of the cDNA sequences according to similarity resulted in six groups (Table S3 in additional file 1). Group *a *was the most divergent and group *d *the most heterogeneous with many allele-specific SNPs. The *Ta *allele apparently resulted from recombination with allele *Sd*. It had been shown previously [26] that Histidine 368 (His368) corresponds to the associated markers *InvGE-6f *and *InvGF-4d*, which are in high linkage disequilibrium with each other due to the close physical linkage between *InvGE *and *InvGF*. The SNP allele *A*_*1103 *_coding for His368 was specific for allele group *a *(Table S3 in additional file 1). The cDNA alleles in *InvGE *group *a *therefore corresponded to the marker *InvGE-6f*. Amplicon sequencing of exon 3 of gene *InvGE *proved difficult due to the presence of the two indels. We therefore amplified and sequenced exon 1 in the ALL population and scored eleven SNPs, which were tested for association with the tuber traits. SNP allele *G*_*95*_, which is diagnostic for alleles *Sa *and *Da*, showed a weak association with CQS, consistent with the association of *InvGE-6f *[25]. One new association was found. The SNP allele *InvGE-A*_*85 *_was positively associated (higher tuber starch content and starch yield) with TSC and TSY (Table 5). Haplotype analysis of 197 individuals using eight diagnostic SNPs in exon 1 identified 19 haplotypes found at frequencies greater than 1% in the ALL population (Table 7). Haplotypes *A *and *B *occurred at frequencies higher than 10% and accounted for 39% of all chromosomes in the population (4 × 197 = 788). Fourteen haplotypes with frequencies between 1% and 10% accounted for 60% of the chromosomes, including the associated alleles *Sa *and *Da*. Six haplotype models were compatible with cDNA sequences, whereas the remaining eleven haplotypes were new.

**Table 7:** InvGE haplotype models obtained with Satlotyper.

Haplotype	**cDNA allele or group **^**1**^	Haplotype frequency	**SNP 85 (*a,d*) **^**2**^	SNP 89 (*x*)	SNP 106 (*Sa, Da*)	SNP 108 (*b*)	SNP 132 (*StinvGE-c*)	SNP 133 (*Tf*)	SNP 135 (*Ta, Sd*)	SNP 162 (*Td*)
*A*	*Se *and *c*	0.265	G	T	A	T	T	G	T	T

*B*	*b*	0.121	G	T	A	A	T	G	T	T

*C*	*Tf*	0.099	G	T	A	T	T	C	T	T

*D*	*Ta *and *d*	0.085	A*	T	A	T	T	G	A	T

*E*		0.057	A*	T	A	A	T	G	T	T

*F*		0.043	G	T	A	T	T	G	A	T

*G*		0.042	A*	T	A	T	T	G	T	T

*H*		0.039	G	T	G*	T	T	G	T	T

*I*		0.037	G	A	A	T	T	C	T	T

*K*		0.027	A*	T	A	A	T	G	A	T

*L*	*Sa *and *Da*	0.025	A*	T	G*	T	T	G	T	T

*M*		0.024	G	T	A	A	T	C	T	T

*N*		0.024	G	A	A	A	T	G	T	T

*O*		0.020	A*	A	A	T	T	G	T	T

*P*		0.019	G	T	G*	A	T	G	T	T

*Q*		0.015	G	A	A	T	A	G	T	T

*R*	*Td*	0.014	A*	T	A	T	T	G	T	G

*S*		0.014	A*	A	A	A	A	G	A	T

*T*		0.012	G	T	A	T	T	G	T	G

^1 ^cDNA allele or allele group that corresponds to the haplotype.

^2 ^cDNA allele or allele group, for which the SNP is diagnostic (see Table S3 in additional file 1).

Associated SNP alleles are highlighted by *.

For *InvGF*, ten cDNA alleles were identified that coded for eight different amino acid sequences (Table 3, Table S4 in additional file 2, Figure S3 in additional file 9). Alignment of the cDNA alleles and *InvGF *from accession AJ133765 (cv Saturna, *StinvGF-b*) [21] revealed 99 SNPs, including three tri-allelic SNPs, which caused amino acid changes at 26 positions, corresponding to 4.5% of the deduced *InvGF *protein. Five similarity groups were distinguished. As in the case of *InvGE*, group *a *was the most divergent and group *d *was the most heterogeneous. The *a *and *d *alleles of *InvGE *and *InvGF *might be part of the same haplotype block. The *InvGF *group *a *alleles are therefore likely to correspond to the marker *InvGF-4d.*

### Natural diversity of InvCD141 and InvCD111 cDNA alleles at the *Inv*_*ap*_*-a *locus

Invertase cDNA alleles at the *Inv*_*ap*_*-a *locus were cloned from leaf tissue. Fewer clones were sequenced than in the case of the loci *Pain-1 *and *Inv*_*ap*_*-b*. Twelve *InvCD141 *cDNA alleles (11 amino acid sequences) were represented among 28 sequences from six genotypes, and 9 *InvCD111 *cDNA alleles (8 amino acid sequences) were obtained from 14 sequences of five genotypes (Table 3). Two additional alleles were found in the database: accessions Z21486 (cv Cara, *StinvCD111-a*) [19] and Z22645 (cv Cara, *StinvCD141-d*) [20]. One hundred and four SNPs (*InvCD141*) including three tri-allelic SNPs, and 71 SNPs (*InvCD111*) caused 32 and 36 amino acid changes, respectively, equivalent to 5-6% protein diversity (Table 3, Tables S5 and S6 in additional files 3 and 4, Figures S4 and S5 in additional files 10 and 11). Grouping of the cDNA alleles according to similarity resulted in six and four groups for *InvCD141 *and *InvCD111*, respectively (Tables S5 and S6 in additional files 3 and 4). Sequencing of the amplified exon 3 of *InvCD141 *in the ALL population allowed us to score 38 SNPs. SNPs specific for the cDNA allele *Sa *(A_280, _T_288, _T_339, _T_543, _A_630, _C_1030, _G_1031, _T_1096_) were all in high linkage disequilibrium with each other. The presence/absence of this *Sa*-specific haplotype (Table S5 in additional file 3) in the ALL population corresponded nearly perfectly to the associated SSCP marker *pCD141-3c *(Jaccard similarity measure 0.92), indicating that the cDNA allele *Sa *corresponds to *pCD141-3c. *Association analysis of the SNPs confirmed *Sa *as an allele that is negatively associated with chip quality and tuber starch content. In addition, one novel, positive association of *InvCD141-G*_*765 *_with CQS, TSC and TSY was detected (Table 5). Haplotype modeling based on 192 individuals and ten SNPs resulted in 18 *InvCD141 *haplotype models (Table 8) with frequencies above 1%. Two haplotypes with frequencies higher than 10% accounted for 27% of all chromosomes in the population (4 × 192 = 768), whereas the remaining 16 haplotypes with frequencies between 1% and 10% accounted for 74% of the chromosomes. Four haplotype models were compatible with cDNA alleles, including the associated allele *Sa *(haplotype *E*), whereas the remaining 14 haplotypes were new.

**Table 8:** InvCD141 haplotype models obtained with Satlotyper.

Haplotype	**cDNA allele or group **^**1**^	Haplotype frequency	**SNP 280 (*Sa*) **^**2**^	SNP 426 (*Sa, Td3*)	SNP 440 (*d*)	SNP 543 (*Sa*)	SNP 673 (*Se*)	SNP 765 (*e*)	SNP 862 (*P18e, Se*)	SNP 889 (*d*)	SNP 1029 (*Sb*)	SNP 1030 (*Sa*)
A	*d*	0.169	G	T	G	C	A	A	A	C	T	G

B	*P54e1, P54e2*	0.101	G	T	C	C	A	G*	A	A	T	G

C		0.086	G	T	C	C	G	A	G	A	T	G

D		0.073	G	T	C	C	G	G*	G	A	G	G

E	*Sa*	0.064	A*	C	C	T*	A	A	A	A	T	C

F		0.060	G	T	G	C	A	G*	A	A	T	G

G	*P18c*	0.060	G	T	C	C	A	A	A	A	T	G

H		0.058	G	T	C	C	G	A	G	A	G	G

I		0.058	G	T	C	C	A	G*	A	C	T	G

K		0.048	G	T	C	C	A	A	G	A	T	G

L		0.047	G	C	C	C	A	A	A	C	T	G

M		0.047	G	C	C	C	G	G*	A	A	G	G

N		0.038	G	T	G	C	G	G*	G	A	G	G

O		0.027	G	T	G	T*	A	A	A	A	T	C

P		0.027	A*	T	C	T*	A	A	A	A	T	C

Q		0.019	G	T	C	C	G	A	A	A	T	G

R		0.017	A*	T	C	T*	G	G*	G	A	T	C

S		0.016	A*	C	G	C	A	A	A	C	T	G

^1 ^cDNA allele or allele group that corresponds to the haplotype.

^2 ^cDNA allele or allele group, for which the SNP is diagnostic (see Table S5 in additional file 3).

Associated SNP alleles are highlighted by *.

#### Phylogenetic analysis of putative invertase proteins

**Figure 2:**
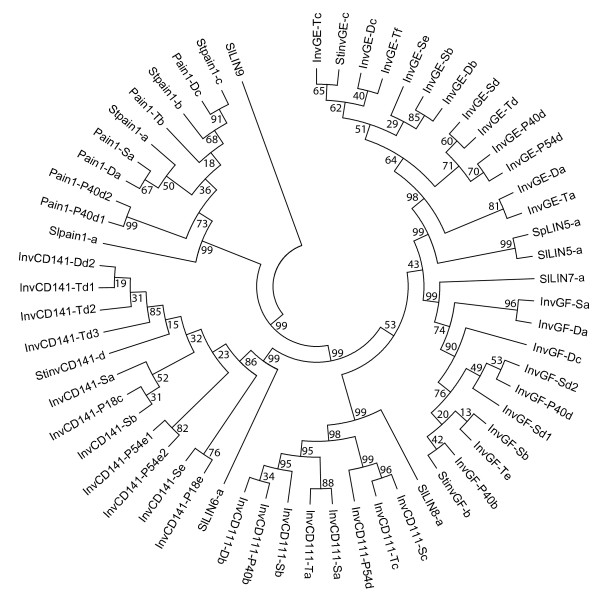
**Phylogenetic tree based on the deduced amino acid sequences of allelic variants of potato and tomato invertase genes**. Forty-six alleles of *Pain1, InvCD111, InvCD141, InvGE *and *InvGF *are described in this study (Tables S2, S3, S4, S5 and S6 in additional files 6, 1, 2, 3 and 4, respectively; Figures S1, S2, S3, S4 and S5 in additional files 7, 8, 9, 10 and 11, respectively). Also included are *Stpain1-a *(AAA50305), *Stpain1-b *(AAQ17074), *Stpain1-c *(ACC93585), *StinvCD141-d *(CAA80358), *StinvGF-b *(CAB76674) and *StinvGE-c *(CAB76673) from *S. tuberosum*, *Slpain1-a *(AAB30874), *SlLIN6-a *(BAA33150), *SlLIN8-a *(AAM28822), *SlLIN7-a *(AAM22410), *SlLIN5-a *(CAB85897) and *SlLIN9 *(CAJ19056) from *S. lycopersicum*, and *SpLIN5-a *(CAB85898) from *S. pennellii*. Bootstrap values are indicated at each branchpoint.

A phylogenetic tree was constructed based on the amino acid sequences deduced from 46 full-length cDNA sequences of *Pain-1*, *InvGE*, *InvGF*, *InvCD141 *and *InvCD111 *(*S. tuberosum*) and seven tomato invertase genes from *S. lycopersicum *and *S. pennellii *(Figure 2). The tree clearly showed five major branches corresponding to the five invertase genes from potato. With the exception of *SlLIN9 *(CAJ19056), which formed a sixth branch, the tomato genes *Slpain1-a *(AAB30874), *SpLIN5-a *(CAB85898), *SlLIN5-a *(CAB85897), *SlLIN7-a *(AAM22410), *SlLIN6-a *(BAA33150) and *SlLIN8-a *(AAM28822) clustered with the respective orthologous potato genes. The interspecific distances between potato and tomato invertases were larger than the intraspecific distances between potato invertase alleles. *Pain-1 *was more closely related to the gene pair *InvCD111/InvCD141 *than to *InvGE/InvGF*.

## Discussion

Analysis of 193 cDNA sequences obtained from three tetraploid and three diploid potato genotypes revealed a high level of natural allelic variation in five potato invertase genes. Fifty-five different full-length cDNA sequences were identified, none of which were previously represented in the databases. Most were genotype specific: only nine were isolated from more than one of the cultivars examined. The average SNP density in cultivated potato is one SNP per 21-24 bp [41,42]. The genes *Pain-1 *and *InvCD111 *fell within this range with one SNP per 24 and 25 bp, respectively. The highest variability, with one SNP per 13 bp, was observed in the *InvGE *gene. *InvGF *and *InvCD141*, both with one SNP per 17 bp, also had higher than average variability. A total of 479 SNPs were detected, and nine (1.6%) were tri-allelic. The 55 identified sequence variants represent a minimum estimate of the number of invertase alleles present in the six genotypes. Other alleles may have been missed due to template bias during PCR amplification [40] or because sample sizes were small, e.g. *InvCD141 *and *InvCD111 *in some genotypes. The sequence variants encode 46 distinct invertase proteins that differ from each other by between 4 and 9%. We took various precautions to eliminate sequence variants resulting from mistakes in reverse transcription or PCR. High-fidelity *Taq *polymerases were used to minimize PCR-derived errors, and cDNAs were cloned from different PCR reactions. Three quarters of the cDNA alleles were supported by at least two cDNA sequences, and alleles were defined based on consensus sequences of all cDNA clones derived from an individual genotype, eliminating singletons (SNPs occurring only once in the multiple sequence alignments). Although we cannot prove that all of the 479 SNPs are authentic, it is highly unlikely that more than a tiny minority of the SNPs observed were generated in the test tube. The generally high level of DNA polymorphism in the genome of *Solanum tuberosum *is well documented [29,33,41,42]. However, very few data are available on the range of natural allelic variation among specific potato genes, particularly across multiple genotypes as studied here. Usually, potato genes are cloned and functionally characterized in a single cultivar. However, five different full-length cDNA clones encoding potato allene oxide synthase 2 (*StAOS2*) have been cloned from three diploid genotypes and functionally characterized [43]. High sequence variability has also been observed among genuine cDNA clones encoding Kunitz-type inhibitors in two tetraploid varieties [44]. The reason for this high genetic plasticity of the cultivated potato can be found in its reproductive biology. Potatoes are clonally propagated on an annual cycle. At much larger intervals (on the order of decades), the vegetative cycle is interrupted by sexual reproduction in the context of breeding programs. These combine heterozygous parental clones, select recombinant seedlings and propagate them again clonally. Switching between clonal and sexual reproduction may have been exploited during potato domestication and evolution, when farmers observed and selected in the field spontaneous hybrid seedlings among their clonally propagated crop [45]. In such a reproductive system, somatic mutations occurring at random during clonal propagation can give rise to cell lineages that eventually enter the germ line. Clonal selection will remove deleterious and favor beneficial somatic mutations [45]. The buffering capacity of a tetraploid genome may also allow the propagation of recessive deleterious mutations. The potato genome therefore represents a rich natural reservoir of mutant genes. In this respect, the potato genome stands in sharp contrast to the genome of its close relative the tomato (*Solanum lycopersicum*). The two genomes are highly colinear, but tomato shows very little intraspecific variation [46]. In contrast to potato, tomato is self-compatible and is propagated exclusively via seeds. Comparative functional characterization of natural potato invertase alleles - which is now possible, promises to uncover some interesting structure-function relationships. Functional differences between the coding regions of potato invertase alleles may be uncovered by the complementation of a yeast invertase mutant and the biochemical characterization of the heterologous expressed proteins [28]. Differences in the expression of alleles can be detected by quantifying the expression based on allele specific SNPs and pyrosequencing technology (Draffehn et al., manuscript in preparation).

The work reported here was designed to identify full-length cDNA clones encoding invertase alleles that are associated with chip quality, tuber starch content and starch yield [25] for further functional analysis. This goal was achieved for the associated markers *Pain1-9a*, *Pain1-8c*, *InvGE-6f/InvGF-4d *and *pCD141-3c*, but not for *Pain1-5b *and *Pain1-5c*, perhaps because an insufficient number of cDNA clones was analyzed. Alternatively, these SSCP markers might result from intron polymorphism that is not detectable in cDNA sequences. The full range of invertase alleles present in a population of 219 tetraploid individuals can be captured by genotyping 15, 11 and 38 SNPs, respectively, at the three invertase loci. Association analysis of the SNPs identified two new associations, one between the SNP allele *InvGE-A*_*85 *_and TSC and TSY, and the other between *InvCD141-G*_*765 *_and CQS, TSC and TSY (Table 5). The *Pain1-a *alleles showed the most significant and positive effects on chip quality and tuber starch content. The corresponding SSCP markers also show epistatic interactions with other candidate loci, which increase starch yield [47]. However, the frequency of these alleles in the ALL population was less than 10%. Enrichment of *Pain1-a *alleles in breeding populations should facilitate the selection of cultivars with improved quality traits. Interestingly, the three SNPs diagnostic for *Pain-1 *alleles of group *a *(*C*_*552*_, *A*_*718 *_and *A*_*1544 *_) differed in their associations. These SNPs are in strong but not absolute linkage disequilibrium with each other. *Pain1-C*_*552 *_and *Pain1*-*A*_*718 *_were more strongly associated with chip quality after cold storage, whereas *Pain1-A*_*1544 *_was mainly associated with tuber starch content. Whereas the cDNA alleles *Pain1-Da, Sa *and *P18a *contained all three SNPs, the allele *StPain1-a *(AAA50305) contained only *A*_*1544*_. This and the four different haplotype models obtained for these three SNPs (haplotypes *K*, *M*, *N*, *O*, Table 6) suggest further structural and possibly functional differentiation between the associated *Pain1-a *alleles, which may be exploitable for marker-assisted selection. *Pain1-snp552 *causes a synonymous nucleotide change, whereas *Pain1-snp718 *and *Pain1-snp1544 *induce non-synonymous changes. *Pain1-SNP1544 *causes the non-conservative amino acid change Thr515Lys. Whether these differences between the coding sequences or differences in the corresponding promoter regions are causal for possibly functional variation remains to be elucidated.

The direct inference of haplotypes from amplicon sequences requires that loci be homozygous, which is rarely the case in diploid and tetraploid potato genotypes. In amplicon sequences derived from heterozygous loci, the phase of the SNPs is unknown. The SATlotyper software was developed to model haplotypes in polyploid species based on unphased SNP data [40]. Haplotype modeling with a subset of the SNPs scored in the invertase genes *Pain-1*, *InvGE *and *InvCD141 *resulted for each gene in two or three haplotypes with higher frequencies (above 10%) and a large number of minor haplotypes with low frequencies. This haplotype distribution might be typical for nuclear genes of the European potato and a signature of its domestication, introduction and breeding history [48-50]. More genes must be analyzed however, before any general conclusions can be drawn. The occurrence of numerous low-frequency haplotypes is compatible with introgressions from other tuber-bearing *Solanum *species during 20^th^-century breeding programs [50] and/or sequence diversification due to fixation of somatic mutations as outlined above. The SNP information will be useful for tracing specific haplotypes back to their origin.

Haplotype modeling correctly predicted some, but not all cDNA alleles that were obtained experimentally from the cvs Satina, Diana and Theresa and could have been detected based on the chosen SNP set. This is because, based on the analysis of sequence trace files, the SNP allele dosage cannot be scored with complete accuracy in all individuals used for haplotype modeling. A small percentage of scoring errors leads to haplotype artefacts with low frequency. To eliminate such artefacts, we considered only haplotype models with a frequency above 1%. As this is an arbitrary threshold, we cannot exclude the possibility that some of the remaining low-frequency haplotypes are also artefacts. Nevertheless, our results demonstrate that SATlotyper is a valuable tool for the fast identification of the major haplotypes present in populations of tetraploid potatoes. The determination of the exact haplotype composition of a tetraploid individual, including rare haplotypes, calls for an allele cloning approach such as that performed in this study.

As in tomato [22], the four genes encoding cell wall-bound acidic invertases in potato are organized in two pairs of tandemly duplicated genes on chromosomes IX and X. The soluble acid invertase is encoded by a single gene on potato chromosome III. The existence of a sixth potato invertase gene is predicted, which is orthologous to the tomato gene *SlLIN9 *located on chromosome 8 (based on the draft genome sequence of tomato provided by "The International Tomato Genome Sequencing Consortium" available at http://solgenomics.net and on personal communication from Gisella Orjeda, Universidad Peruana Cayetano Heredia, Peru). This gene might encode a soluble neutral invertase not characterized so far in potato. The five characterized potato invertase genes are all located within segmental chromosome duplications of unknown size, which show structural conservation with other, distantly related plant species [51,52]. Functionally essential parts of an ancestral plant genome might be preserved in such conserved chromosome segments. The important role invertases play in many aspects of plant life is consistent with their location in evolutionarily ancient parts of plant genomes.

## Conclusions

Very high natural allelic variation in five potato invertase genes was uncovered by sequence analysis of full length cDNA clones from six different genotypes and SNP analysis in a larger association mapping population. This variability is explained by the potato's reproductive biology. Some of the structural variation found might be causal for functional variation, which influences important agronomic traits of the potato such as tuber starch and sugar content. The invertase cDNA clones described here are the basis for further functional studies. The associations found between specific invertase alleles and tuber starch content, starch yield and chip quality facilitate the selection of superior potato genotypes in breeding programs. Finally, our results point out that natural variation should be taken into account when conducting molecular and functional characterization of potato genes.

## List of abbreviations

ASA: allele specific amplification; BAC: Bacterial artificial chromosome; CQA: Chip quality in autumn; CQS: Chip quality after storage at 4°C; LD: linkage disequilibrium; PCR: Polymerase chain reaction; QTL: Quantitative trait locus; SCAR: sequence characterized amplified region; SSCP: single strand conformation polymorphism; TSC: tuber starch content; TSY: tuber starch yield; TY: tuber yield.

## Authors' contributions

AD carried out the cDNA cloning, the sequence analyses and the haplotype modeling, and participated in the SNP analysis. SM performed the SNP analysis and contributed to the association statistics and haplotype modeling. LL carried out the pyrosequencing. CG conceived the study, participated in its design and coordination, carried out statistical analyses and drafted the manuscript. All authors read and approved the final manuscript.

## Supplementary Material

Additional file 1 **Table S3: SNPs differentiating *InvGE *cDNA alleles**. Click here for file

Additional file 2 **Table S4: SNPs differentiating *InvGF *cDNA alleles**. Click here for file

Additional file 3 **Table S5: SNPs differentiating *InvCD141 *cDNA alleles**. Click here for file

Additional file 4 **Table S6: SNPs differentiating *InvCD111 *cDNA alleles**. Click here for file

Additional file 5 **Table S1: Annotation of BAC clones BC149o15 and BC163l15**. Click here for file

Additional file 6 **Table S2: SNPs differentiating *Pain-1 *cDNA alleles**. Click here for file

Additional file 7 **Figure S1: Amino acid alignment of *Pain-1 *cDNA alleles**. Click here for file

Additional file 8 **Figure S2: Amino acid alignment of *InvGE *cDNA alleles**. Click here for file

Additional file 9 **Figure S3: Amino acid alignment of *InvGF *cDNA alleles**. Click here for file

Additional file 10 **Figure S4: Amino acid alignment of *InvCD141 *cDNA alleles**. Click here for file

Additional file 11 **Figure S5: Amino acid alignment of *InvCD111 *cDNA alleles**. Click here for file
