# Supplementary material for: Natural diversity of potato (Solanum tuberosum) invertases
Source: BMC Plant Biol. 2010 Dec 9;10:271. doi: 10.1186/1471-2229-10-271 (PMC3012049; doi:10.1186/1471-2229-10-271)
Supplement: Additional file 5 — Table S1: Annotation of BAC clones BC149o15 and BC163l15. [file 1471-2229-10-271-S5.DOC]

**Supplementary Table 1**: Annotation of BAC clones BC149o15 and BC163l15.

| BAC | Gene number ^1^  (strand) | Position [bp] | Description |
| --- | --- | --- | --- |
| BC149o15 | 1 (+) | 2967-7658 | Putative retrotransposon protein |
|  | 2 (+) | 9046-10,325 | Unknown |
|  | 3 (+) | 24,790-26,753 | Putative transposon protein |
|  | 4 (+) | 27,294-28,747 | Putative transposase |
|  | 5 (-) | 54,835-50,885 | Invertase *Pain-1*, β-fructofuranosidase |
|  | 6 (+) | 68,295-72,570 | Putative retroelement polyprotein |
| BC163l15 | 1 (+) | 2,068-2,454 | Auxin responsive gene |
|  | 2 (+) | 4,655-13,035 | Kinesin |
|  | 3 (+) | 31,961-36,971 | Cell wall invertase *InvCD111*, β-fructofuranosidase |
|  | 4 (+) | 44,311-47,793 | Cell wall invertase *InvCD141*, β-fructofuranosidase |
|  | 5 (-) | 53,022-50,302 | Putative ribosomal protein |
|  | 6 (-) | 57,824-54,722 | Putative esterase lipase |
|  | 7 (+) | 61,479-63,535 | GTP-binding protein |
|  | 8 (-) | 67,349-65,377 | Putative dynamin |
|  | 9 (+) | 68,953-70,012 | Unknown |
|  | 10 (+) | 72,520-73,558 | Putative integral membrane family protein |
|  | 11 (+) | 74,343-77,806 | Putative RNA-binding protein |
|  | 12 (-) | 80,608-79,442 | Unknown |
|  | 13 (+) | 84,626-87,464 | Putative embryo defective protein |
|  | 14 (-) | 93,506-88,268 | DNA-binding protein |
|  | 15 (-) | 107,512-104,330 | Pre-mRNA splicing factor |

^1^ Gene numbering is according to Figure 1
