# Supplementary material for: Natural diversity of potato (Solanum tuberosum) invertases
Source: BMC Plant Biol. 2010 Dec 9;10:271. doi: 10.1186/1471-2229-10-271 (PMC3012049; doi:10.1186/1471-2229-10-271)
Supplement: Additional file 7 — Figure S1: Amino acid alignment of Pain-1 cDNA alleles. [file 1471-2229-10-271-S7.DOC]

**Supplementary Figure 1**: Amino acid alignment of six new *Pain-1* cDNA alleles (*Pain1-Da, Pain1-Sa, Pain1-Dc, Pain1-Tb, Pain1-P40d1, Pain1-P40d2*) and gene bank accessions AAA50305 (*Stpain1_a*), AAQ17074 (*Stpain1_b*), ACC93585 (*Stpain1_c*), of *S. tuberosum*, and AAB30874 (*Slpain1_a*), AAL75450 (*Slpain1_b*), 1905419A (*Slpain1_c*) of *S. lycopersicum*. Amino acid positions that distinguish potato (*S. tuberosum*) and tomato (*S. lycopersicum*) are highlighted in red versus yellow. All other polymorphic amino acids are shown in green versus grey.
