# Supplementary material for: Natural diversity of potato (Solanum tuberosum) invertases
Source: BMC Plant Biol. 2010 Dec 9;10:271. doi: 10.1186/1471-2229-10-271 (PMC3012049; doi:10.1186/1471-2229-10-271)
Supplement: Additional file 8 — Figure S2: Amino acid alignment of InvGE cDNA alleles. [file 1471-2229-10-271-S8.DOC]

**Supplementary Figure 2**: Amino acid alignment of 12 new *InvGE* cDNA alleles and gene bank accessions CAB76673 (*StinvGE-c*) of *S. tuberosum*, CAB85898 (*SpLIN5-a*) of *S. pennellii*, and CAB85897 (*SlLIN5-a*), AAO45697 (*SlLIN5-b*) of *S. lycopersicum*. Amino acid positions that distinguish potato (*S. tuberosum*) and tomato (*S. lycopersicum, S. pennellii*), are highlighted in red versus yellow. All other polymorphic amino acids are shown in green versus grey.
