# Supplementary material for: Natural diversity of potato (Solanum tuberosum) invertases
Source: BMC Plant Biol. 2010 Dec 9;10:271. doi: 10.1186/1471-2229-10-271 (PMC3012049; doi:10.1186/1471-2229-10-271)
Supplement: Additional file 9 — Figure S3: Amino acid alignment of InvGF cDNA alleles. [file 1471-2229-10-271-S9.DOC]

**Supplementary Figure 3**: Amino acid alignment of nine new *InvGF* alleles and gene bank accessions CAB76674 (*StinvGF-b*) of potato, and AAM22410 (*SlLIN7-a*) and AAO45698 (*SlLIN7-b*) of tomato. Amino acid positions that distinguish potato (*S. tuberosum*) and tomato (*S. lycopersicum*) are highlighted in red versus yellow. All other polymorphic amino acids are shown in green versus grey.
