# Supplementary material for: Natural diversity of potato (Solanum tuberosum) invertases
Source: BMC Plant Biol. 2010 Dec 9;10:271. doi: 10.1186/1471-2229-10-271 (PMC3012049; doi:10.1186/1471-2229-10-271)
Supplement: Additional file 10 — Figure S4: Amino acid alignment of InvCD141 cDNA alleles. [file 1471-2229-10-271-S10.DOC]

**Supplementary Figure 4**: Amino acid alignment of 11 new *InvCD141* cDNA alleles and gene bank accessions CAA80358 (*StCD141-d*) of *S. tuberosum*, and BAA33150 (*SlLIN6-a*), AAM28823 (*SlLIN6-b*) of *S. lycopersicum*. Amino acid positions that distinguish potato (*S. tuberosum*) and tomato (*S. lycopersicum*) are highlighted in red versus yellow. All other polymorphic amino acids are shown in green versus grey.
