# Supplementary material for: Natural diversity of potato (Solanum tuberosum) invertases
Source: BMC Plant Biol. 2010 Dec 9;10:271. doi: 10.1186/1471-2229-10-271 (PMC3012049; doi:10.1186/1471-2229-10-271)
Supplement: Additional file 11 — Figure S5: Amino acid alignment of InvCD111 cDNA alleles. [file 1471-2229-10-271-S11.DOC]

**Supplementary Figure 5**: Amino acid alignment of eight new *InvCD111* cDNA alleles and gene bank accessions CAA79676 (*StCD111-a*) of *S. tuberosum* and AAM28822 (*SlLIN8-a*) of *S. lycopersicum*. Amino acid positions that distinguish between potato (*S. tuberosum*), and tomato (*S. lycopersicum*), are highlighted in red versus yellow. All other polymorphic amino acids are shown in green versus grey.
